# Supplementary material for: Blood-based epigenome-wide analyses of 19 common disease states: A longitudinal, population-based linked cohort study of 18,413 Scottish individuals
Source: PLoS Med. 2023 Jul 6;20(7):e1004247. doi: 10.1371/journal.pmed.1004247 (PMC10325072; doi:10.1371/journal.pmed.1004247)
Supplement: S2 Appendix — Outliers are highlighted in green (COPD) and blue (type 2 diabetes). (PDF) [file pmed.1004247.s003.pdf]

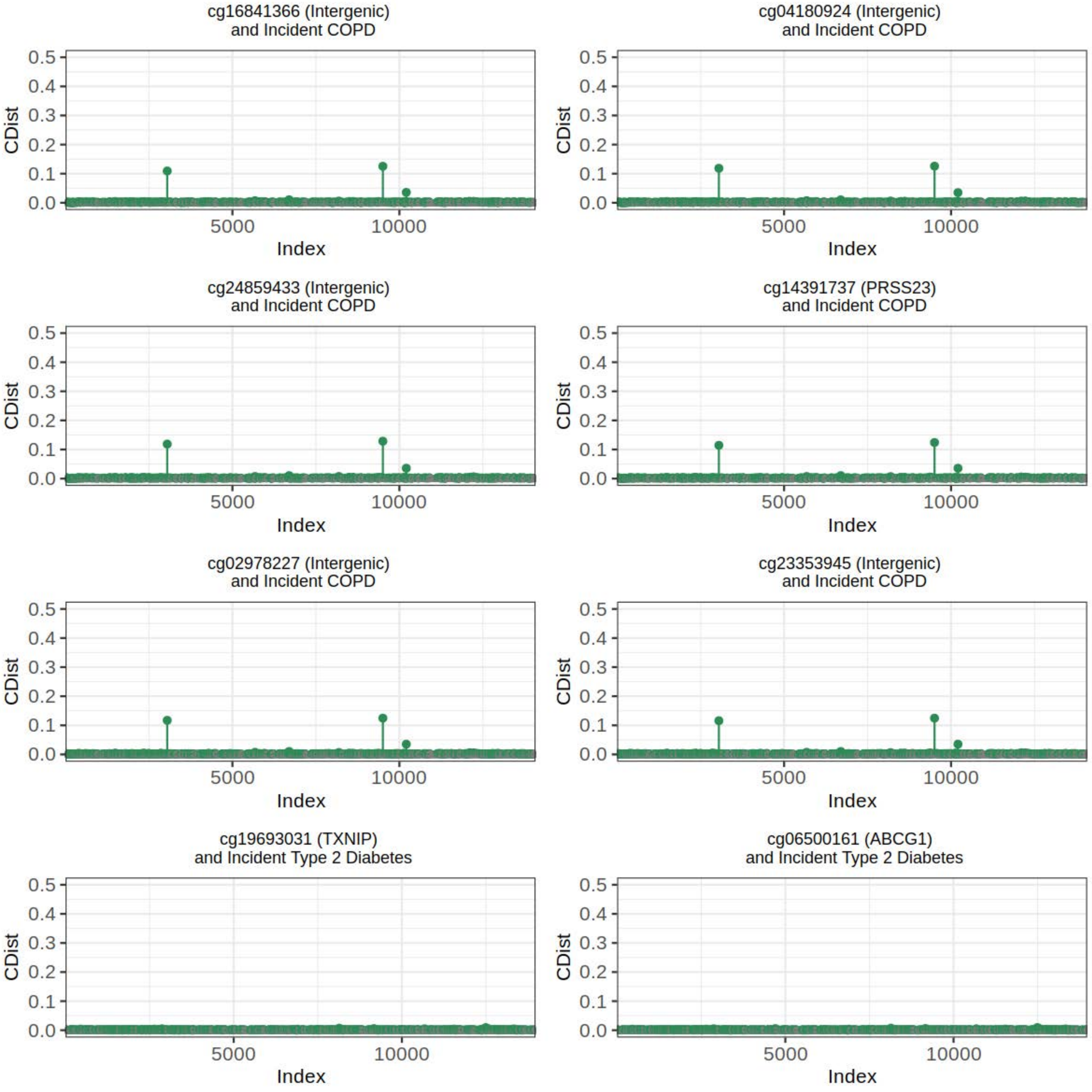

cg16841366 (Intergenic) and Incident COPD
cg04180924 (Intergenic) and Incident COPD
cg24859433 (Intergenic) and Incident COPD
cg14391737 (PRSS23) and Incident COPD
cg02978227 (Intergenic) and Incident COPD
cg23353945 (Intergenic) and Incident COPD
cg19693031 (TXNIP) and Incident Type 2 Diabetes
cg06500161 (ABCG1) and Incident Type 2 Diabetes
CDist
0.0
0.1
0.2
0.3
0.4
0.5
Index
5000
10000

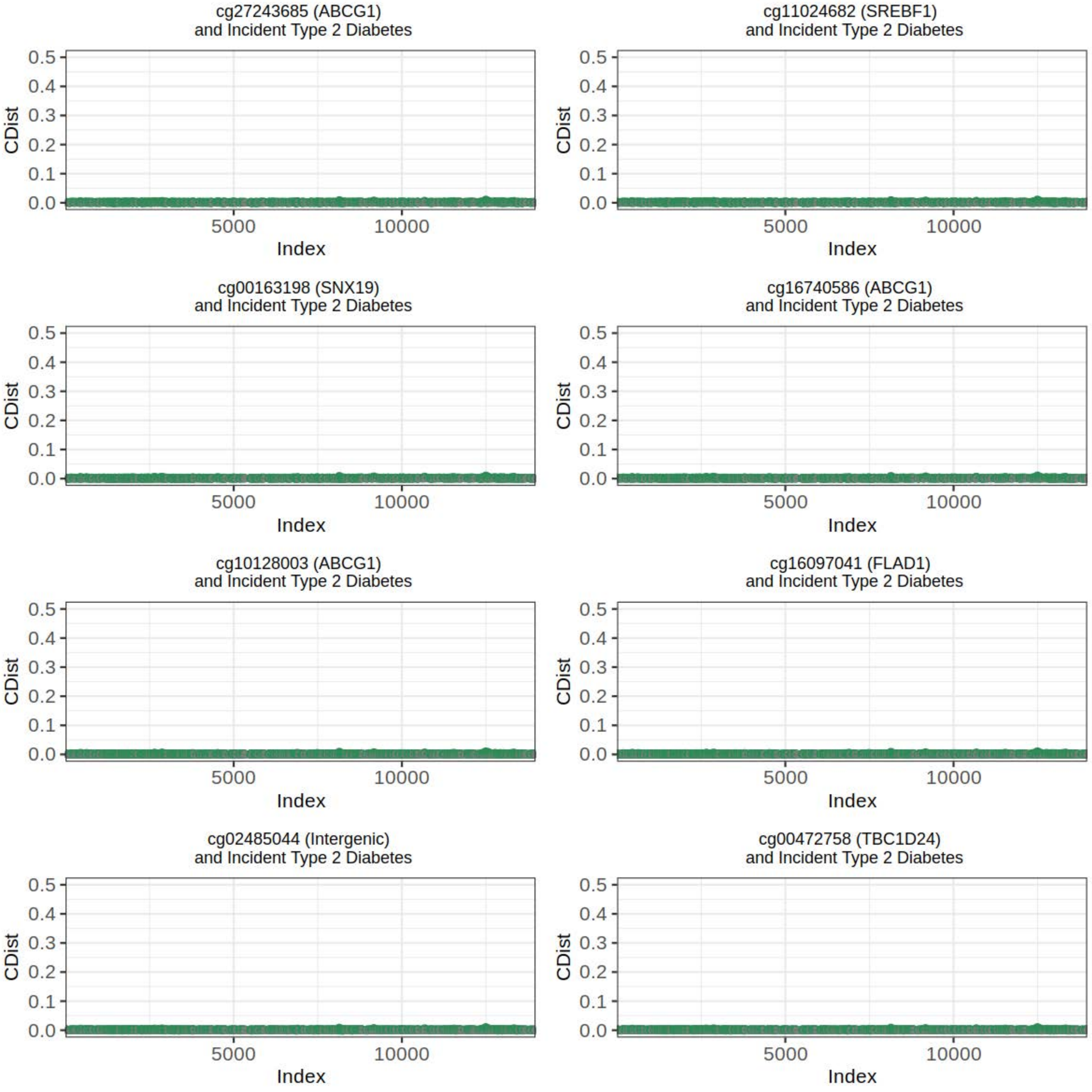
cg27243685 (ABCG1)
and Incident Type 2 Diabetes
cg11024682 (SREBF1)
and Incident Type 2 Diabetes
cg00163198 (SNX19)
and Incident Type 2 Diabetes
cg16740586 (ABCG1)
and Incident Type 2 Diabetes
cg10128003 (ABCG1)
and Incident Type 2 Diabetes
cg16097041 (FLAD1)
and Incident Type 2 Diabetes
cg02485044 (Intergenic)
and Incident Type 2 Diabetes
cg00472758 (TBC1D24)
and Incident Type 2 Diabetes
CDist
0.5
0.4
0.3
0.2
0.1
0.0
Index
5000
10000

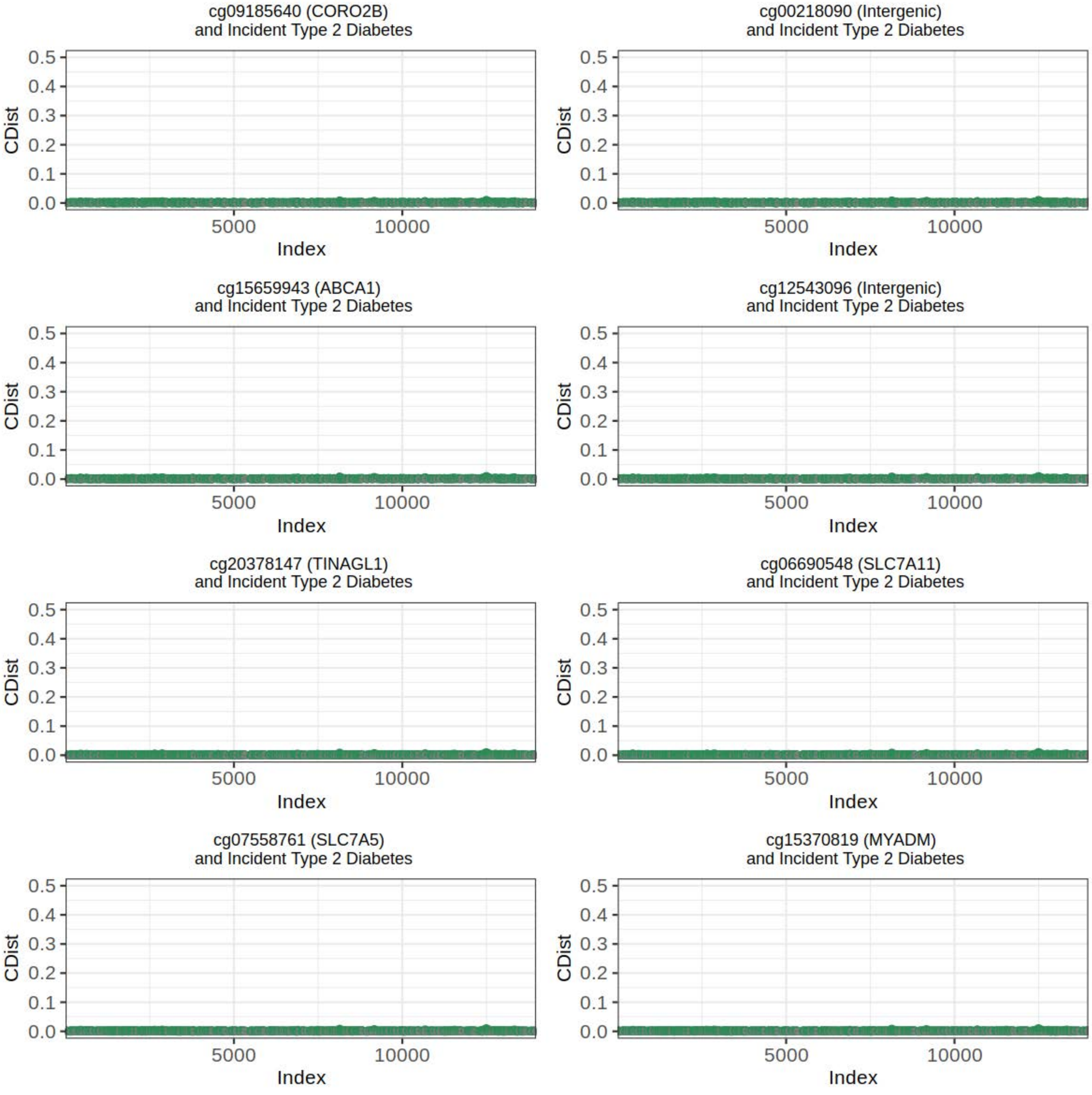
cg09185640 (CORO2B)
and Incident Type 2 Diabetes
cg00218090 (Intergenic)
and Incident Type 2 Diabetes
cg15659943 (ABCA1)
and Incident Type 2 Diabetes
cg12543096 (Intergenic)
and Incident Type 2 Diabetes
cg20378147 (TINAGL1)
and Incident Type 2 Diabetes
cg06690548 (SLC7A11)
and Incident Type 2 Diabetes
cg07558761 (SLC7A5)
and Incident Type 2 Diabetes
cg15370819 (MYADM)
and Incident Type 2 Diabetes
CDist
Index
0.0
0.1
0.2
0.3
0.4
0.5
5000
10000

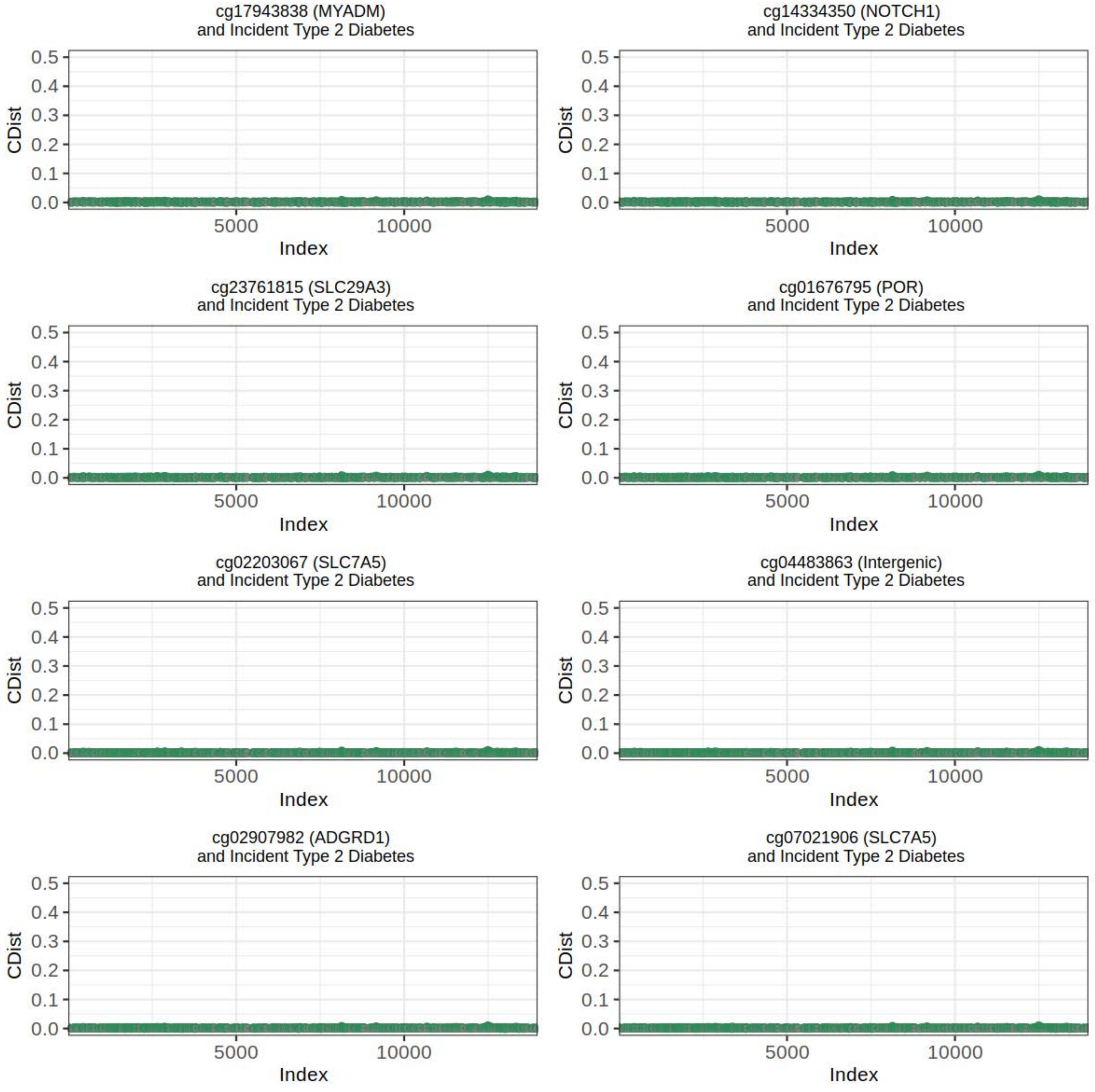

cg17943838 (MYADM)
and Incident Type 2 Diabetes
cg14334350 (NOTCH1)
and Incident Type 2 Diabetes
cg23761815 (SLC29A3)
and Incident Type 2 Diabetes
cg01676795 (POR)
and Incident Type 2 Diabetes
cg02203067 (SLC7A5)
and Incident Type 2 Diabetes
cg04483863 (Intergenic)
and Incident Type 2 Diabetes
cg02907982 (ADGRD1)
and Incident Type 2 Diabetes
cg07021906 (SLC7A5)
and Incident Type 2 Diabetes
CDist
0.5
0.4
0.3
0.2
0.1
0.0
5000
10000
Index

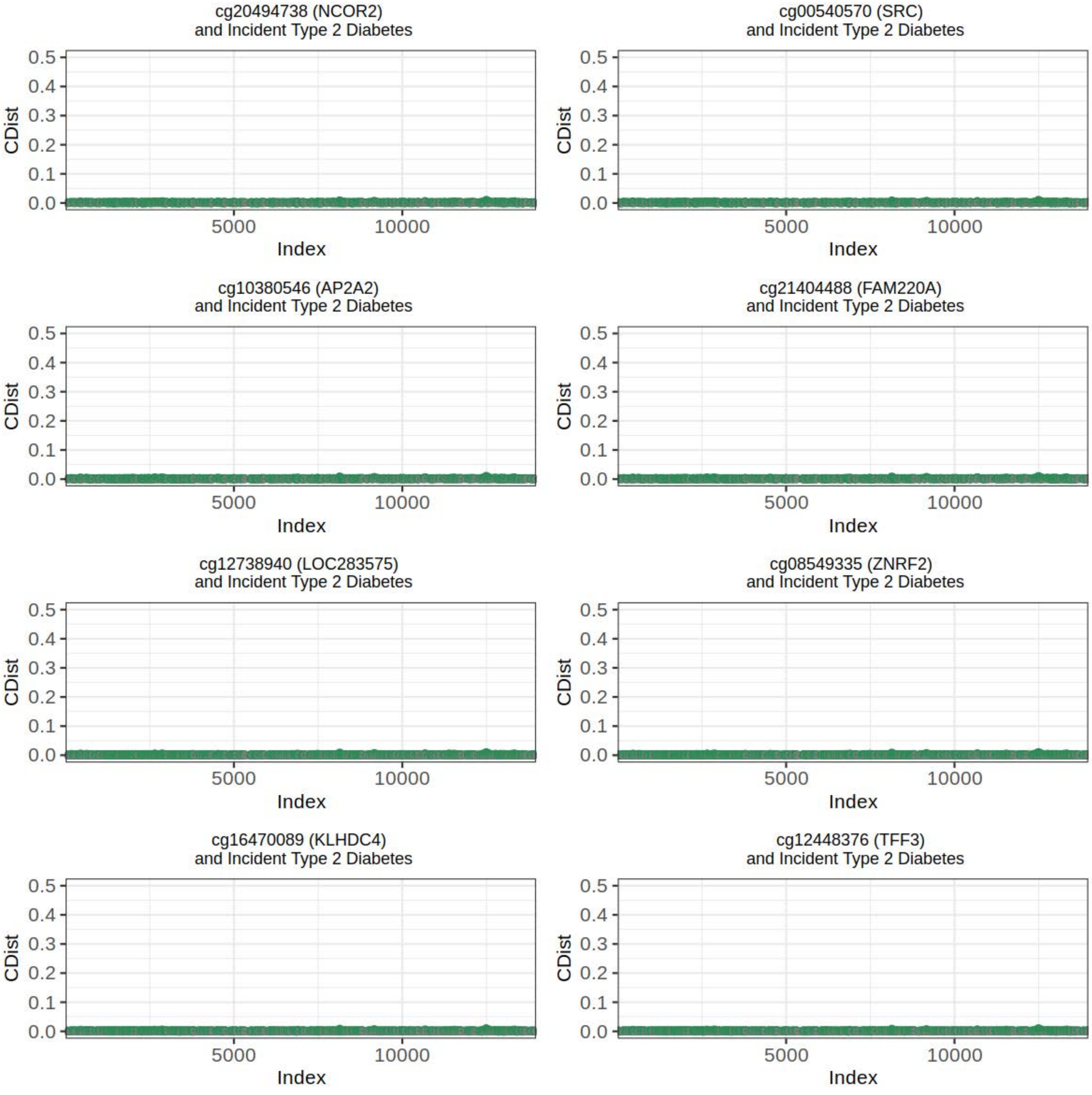
cg20494738 (NCOR2)
and Incident Type 2 Diabetes
cg00540570 (SRC)
and Incident Type 2 Diabetes
cg10380546 (AP2A2)
and Incident Type 2 Diabetes
cg21404488 (FAM220A)
and Incident Type 2 Diabetes
cg12738940 (LOC283575)
and Incident Type 2 Diabetes
cg08549335 (ZNRF2)
and Incident Type 2 Diabetes
cg16470089 (KLHDC4)
and Incident Type 2 Diabetes
cg12448376 (TFF3)
and Incident Type 2 Diabetes
CDist
Index
0.0
0.1
0.2
0.3
0.4
0.5
5000
10000

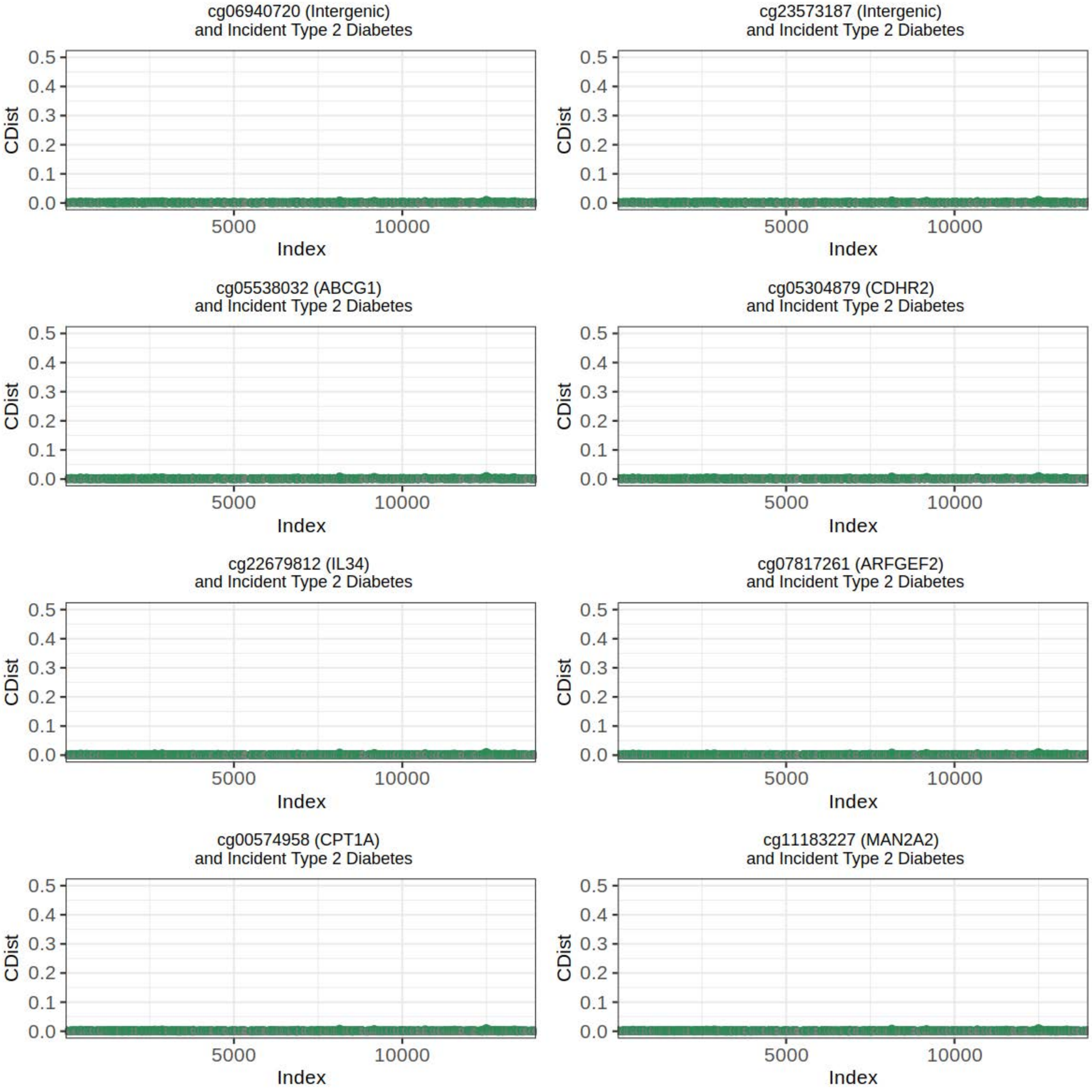
cg06940720 (Intergenic)
and Incident Type 2 Diabetes
cg23573187 (Intergenic)
and Incident Type 2 Diabetes
cg05538032 (ABCG1)
and Incident Type 2 Diabetes
cg05304879 (CDHR2)
and Incident Type 2 Diabetes
cg22679812 (IL34)
and Incident Type 2 Diabetes
cg07817261 (ARFGEF2)
and Incident Type 2 Diabetes
cg00574958 (CPT1A)
and Incident Type 2 Diabetes
cg11183227 (MAN2A2)
and Incident Type 2 Diabetes
CDist
0.0
0.1
0.2
0.3
0.4
0.5
Index
5000
10000

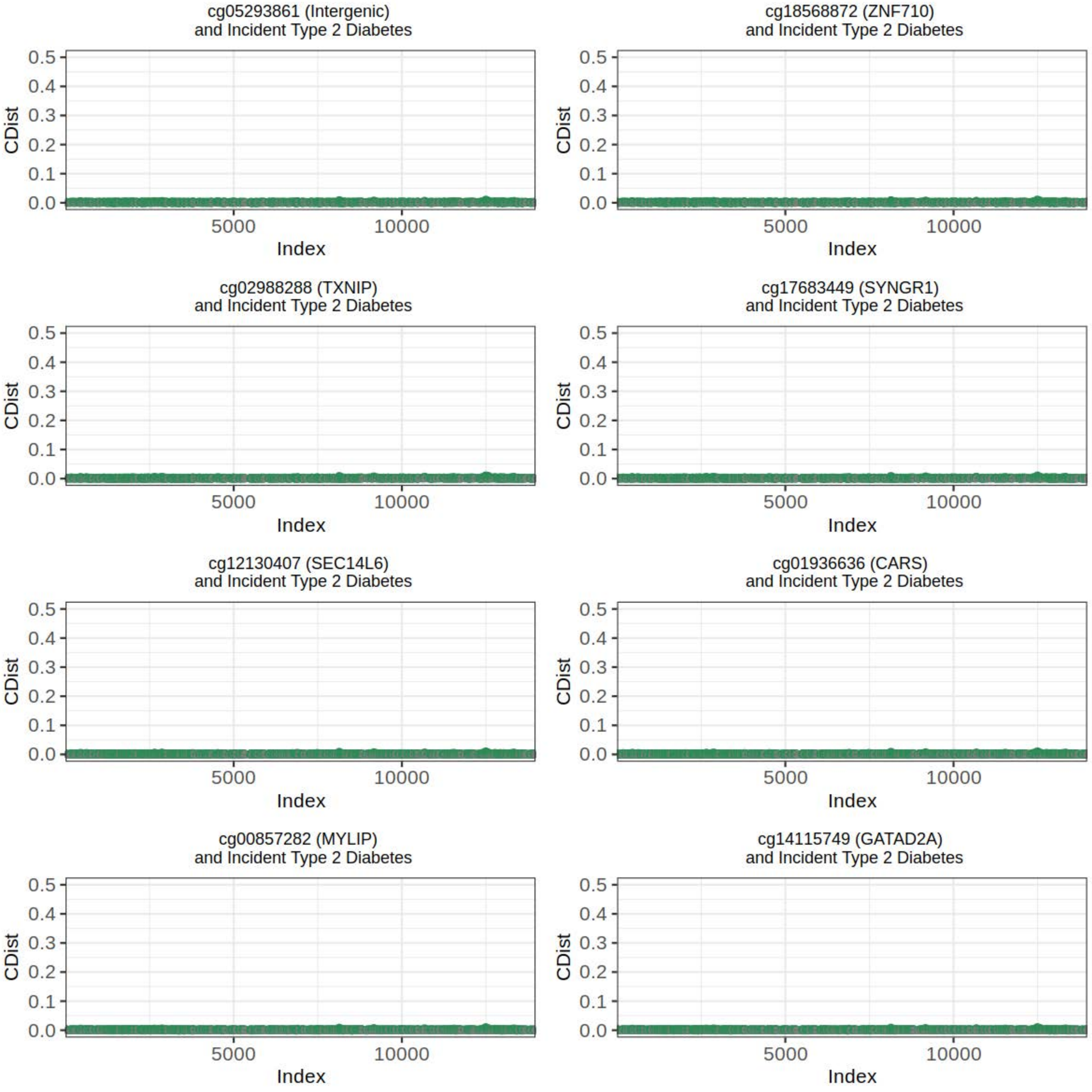
cg05293861 (Intergenic)
and Incident Type 2 Diabetes
cg18568872 (ZNF710)
and Incident Type 2 Diabetes
cg02988288 (TXNIP)
and Incident Type 2 Diabetes
cg17683449 (SYNGR1)
and Incident Type 2 Diabetes
cg12130407 (SEC14L6)
and Incident Type 2 Diabetes
cg01936636 (CARS)
and Incident Type 2 Diabetes
cg00857282 (MYLIP)
and Incident Type 2 Diabetes
cg14115749 (GATAD2A)
and Incident Type 2 Diabetes
CDist
0.5
0.4
0.3
0.2
0.1
0.0
5000
10000
Index

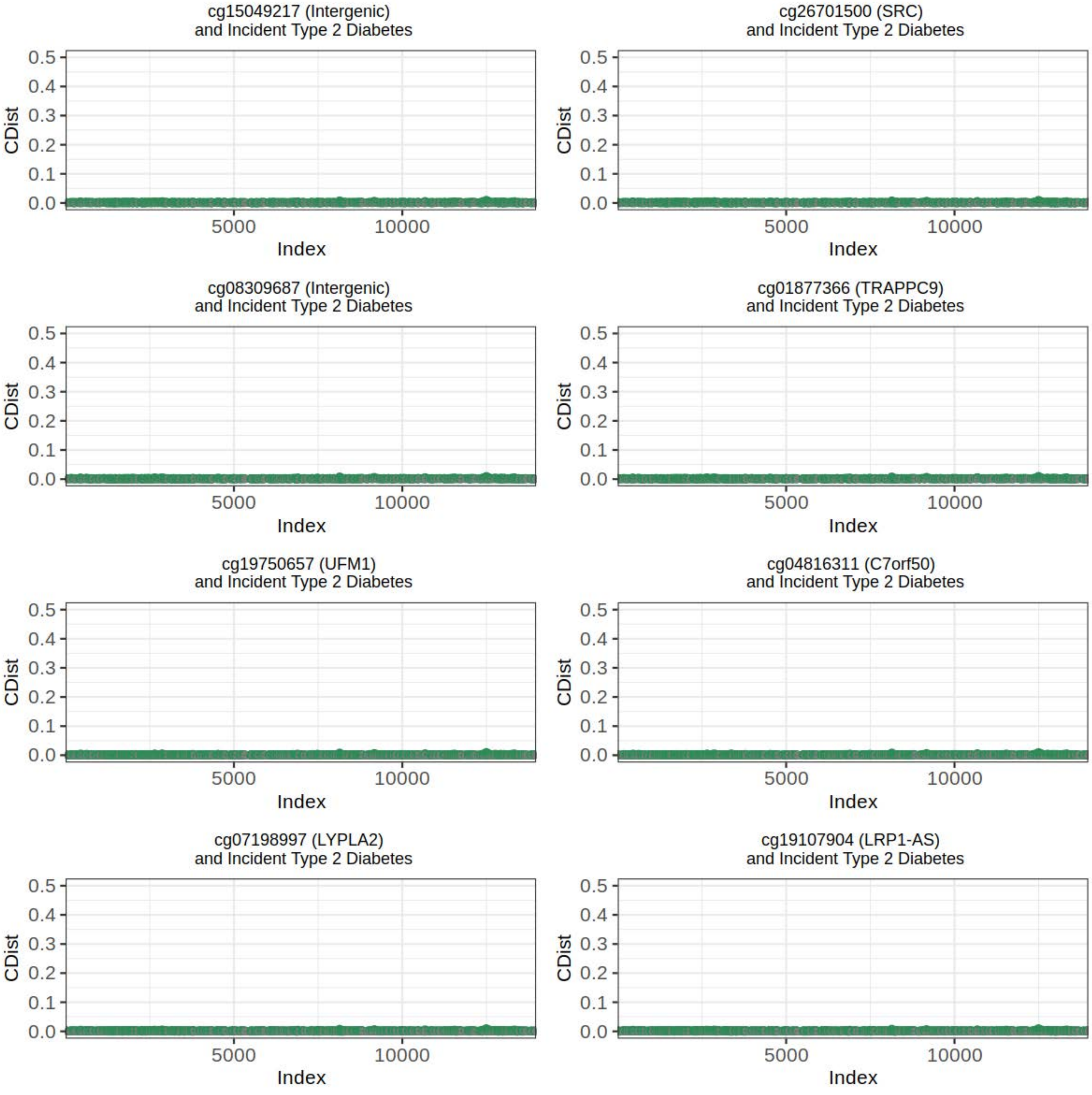
cg15049217 (Intergenic)
and Incident Type 2 Diabetes
CDist
Index
cg26701500 (SRC)
and Incident Type 2 Diabetes
CDist
Index
cg08309687 (Intergenic)
and Incident Type 2 Diabetes
CDist
Index
cg01877366 (TRAPPC9)
and Incident Type 2 Diabetes
CDist
Index
cg19750657 (UFM1)
and Incident Type 2 Diabetes
CDist
Index
cg04816311 (C7orf50)
and Incident Type 2 Diabetes
CDist
Index
cg07198997 (LYPLA2)
and Incident Type 2 Diabetes
CDist
Index
cg19107904 (LRP1-AS)
and Incident Type 2 Diabetes
CDist
Index
0.0
0.1
0.2
0.3
0.4
0.5
5000
10000

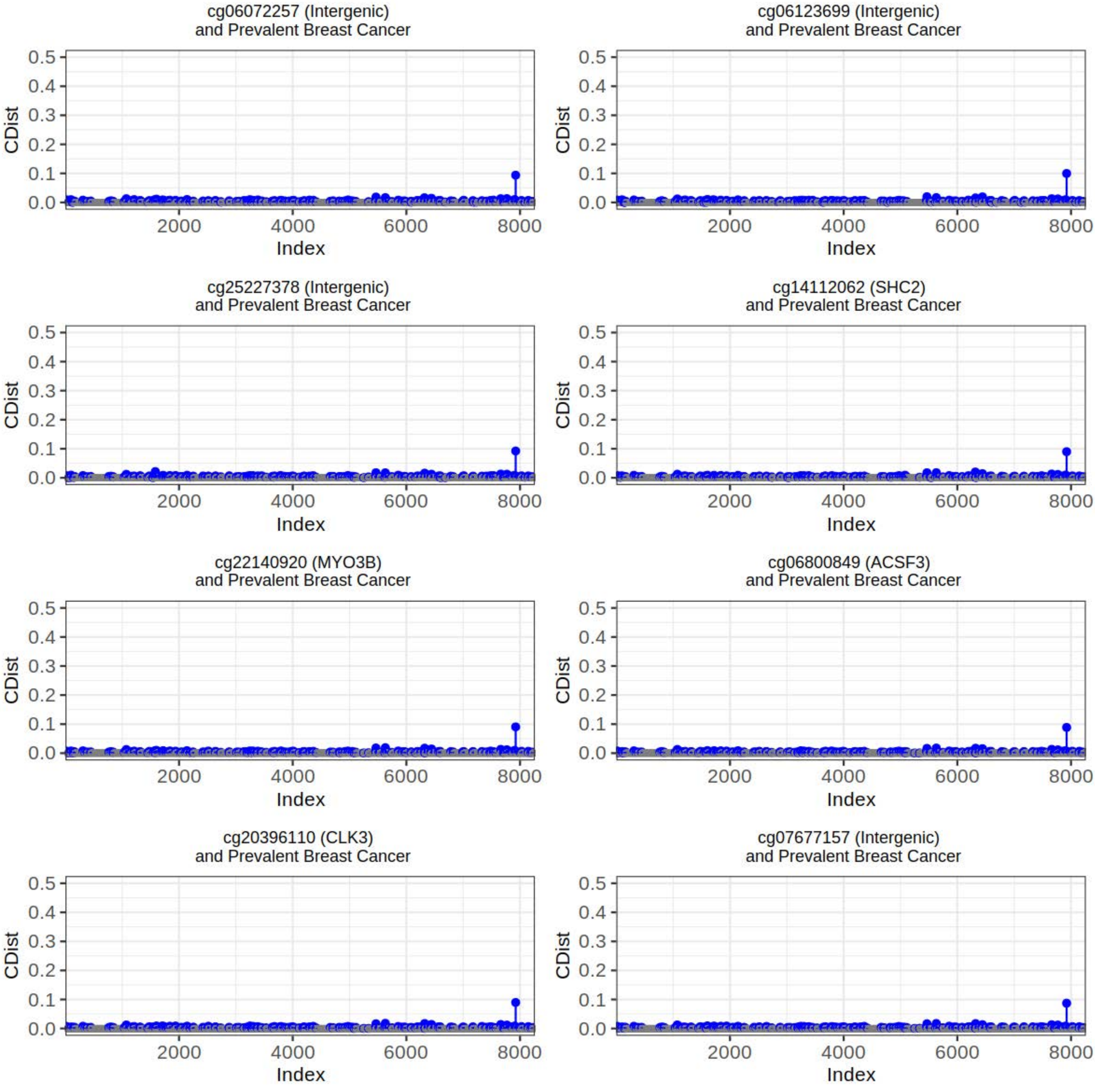
cg06072257 (Intergenic)
and Prevalent Breast Cancer
cg06123699 (Intergenic)
and Prevalent Breast Cancer
cg25227378 (Intergenic)
and Prevalent Breast Cancer
cg14112062 (SHC2)
and Prevalent Breast Cancer
cg22140920 (MYO3B)
and Prevalent Breast Cancer
cg06800849 (ACSF3)
and Prevalent Breast Cancer
cg20396110 (CLK3)
and Prevalent Breast Cancer
cg07677157 (Intergenic)
and Prevalent Breast Cancer
CDist
0.0
0.1
0.2
0.3
0.4
0.5
Index
2000
4000
6000
8000

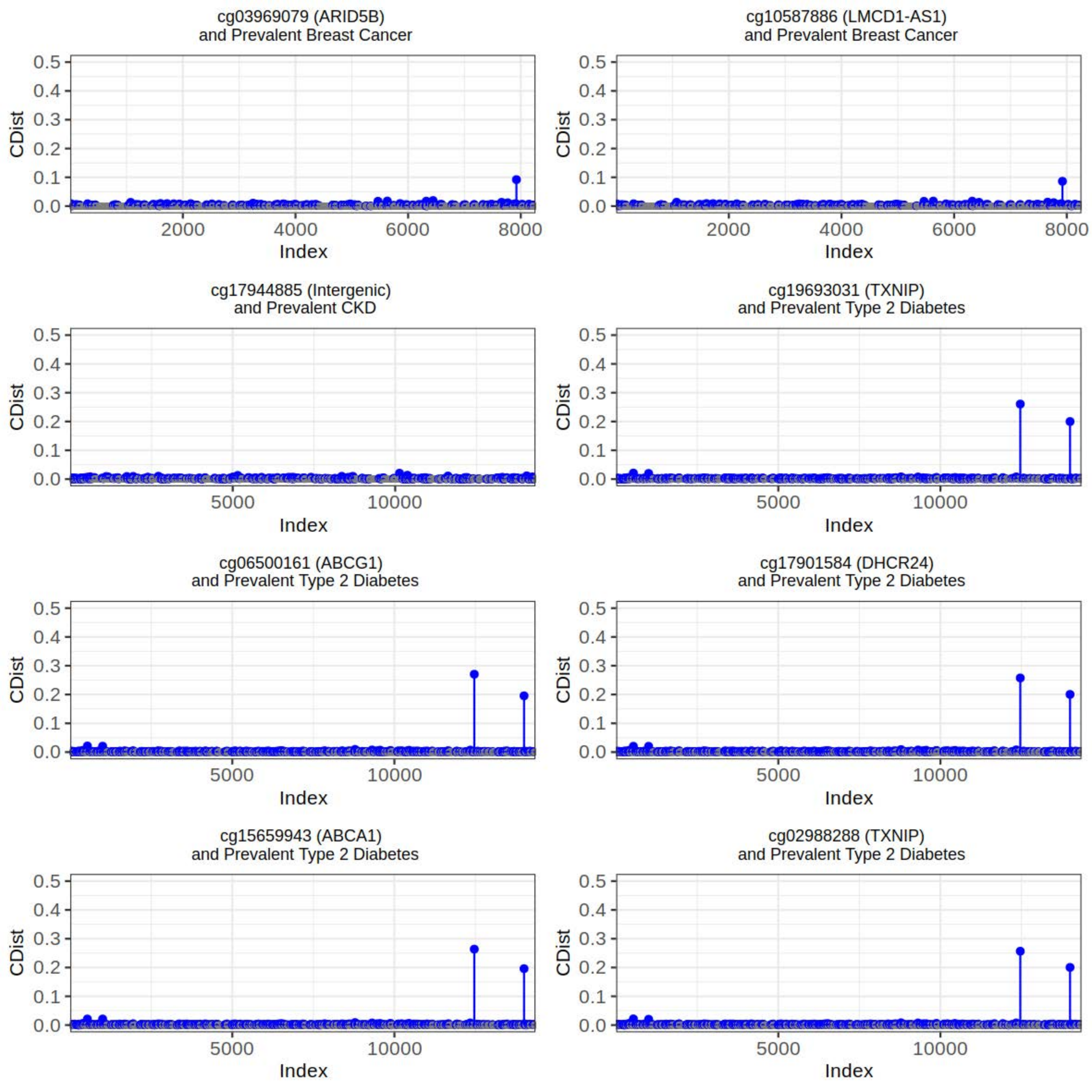
cg03969079 (ARID5B)
and Prevalent Breast Cancer
cg10587886 (LMCD1-AS1)
and Prevalent Breast Cancer
cg17944885 (Intergenic)
and Prevalent CKD
cg19693031 (TXNIP)
and Prevalent Type 2 Diabetes
cg06500161 (ABCG1)
and Prevalent Type 2 Diabetes
cg17901584 (DHCR24)
and Prevalent Type 2 Diabetes
cg15659943 (ABCA1)
and Prevalent Type 2 Diabetes
cg02988288 (TXNIP)
and Prevalent Type 2 Diabetes
CDist
Index
0.0
0.1
0.2
0.3
0.4
0.5
2000
4000
6000
8000
5000
10000

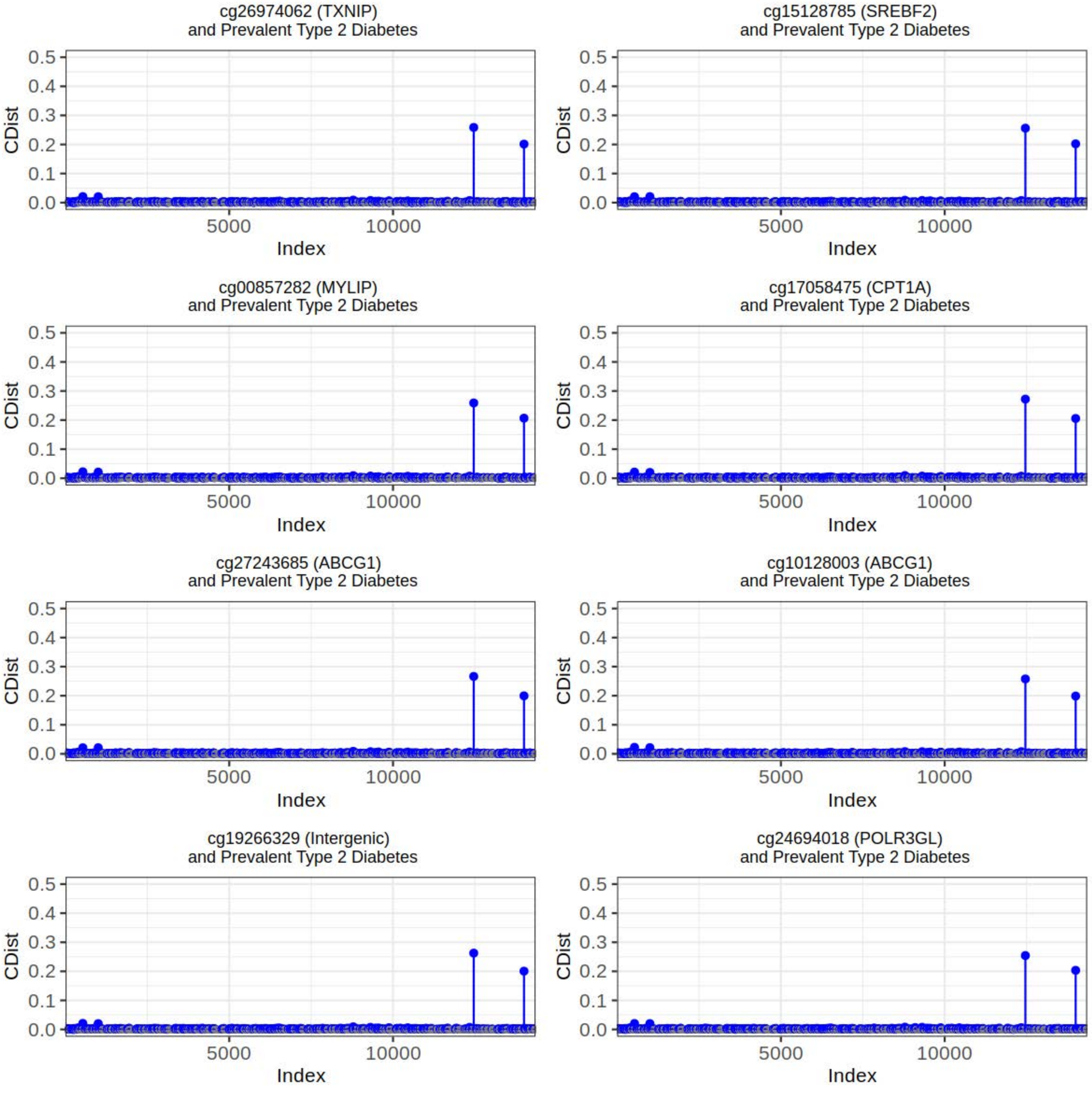
cg26974062 (TXNIP)
and Prevalent Type 2 Diabetes
cg15128785 (SREBF2)
and Prevalent Type 2 Diabetes
cg00857282 (MYLIP)
and Prevalent Type 2 Diabetes
cg17058475 (CPT1A)
and Prevalent Type 2 Diabetes
cg27243685 (ABCG1)
and Prevalent Type 2 Diabetes
cg10128003 (ABCG1)
and Prevalent Type 2 Diabetes
cg19266329 (Intergenic)
and Prevalent Type 2 Diabetes
cg24694018 (POLR3GL)
and Prevalent Type 2 Diabetes
CDist
0.0
0.1
0.2
0.3
0.4
0.5
Index
5000
10000

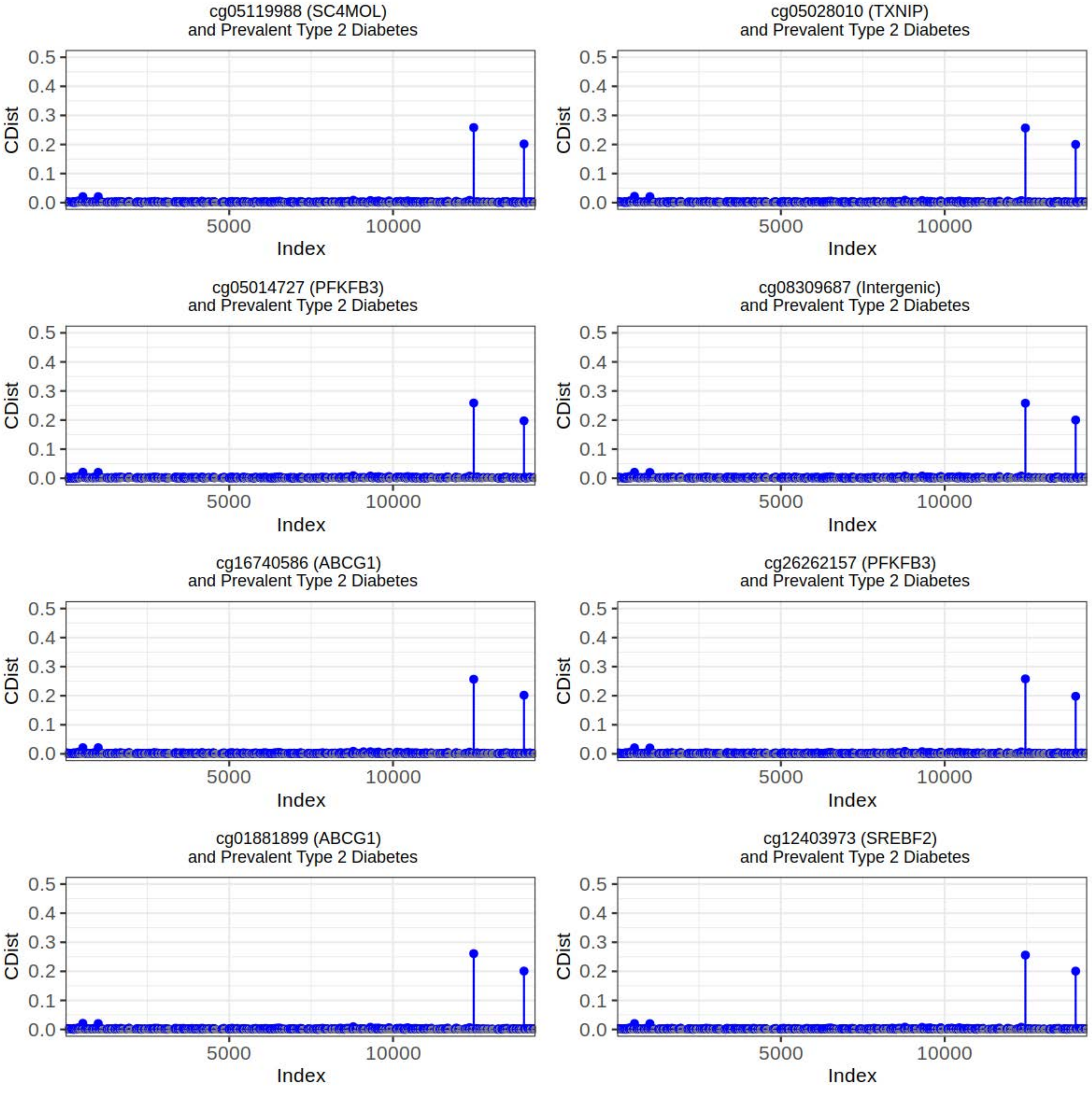

cg05119988 (SC4MOL)
and Prevalent Type 2 Diabetes
cg05028010 (TXNIP)
and Prevalent Type 2 Diabetes
cg05014727 (PFKFB3)
and Prevalent Type 2 Diabetes
cg08309687 (Intergenic)
and Prevalent Type 2 Diabetes
cg16740586 (ABCG1)
and Prevalent Type 2 Diabetes
cg26262157 (PFKFB3)
and Prevalent Type 2 Diabetes
cg01881899 (ABCG1)
and Prevalent Type 2 Diabetes
cg12403973 (SREBF2)
and Prevalent Type 2 Diabetes
CDist
0.0
0.1
0.2
0.3
0.4
0.5
Index
5000
10000

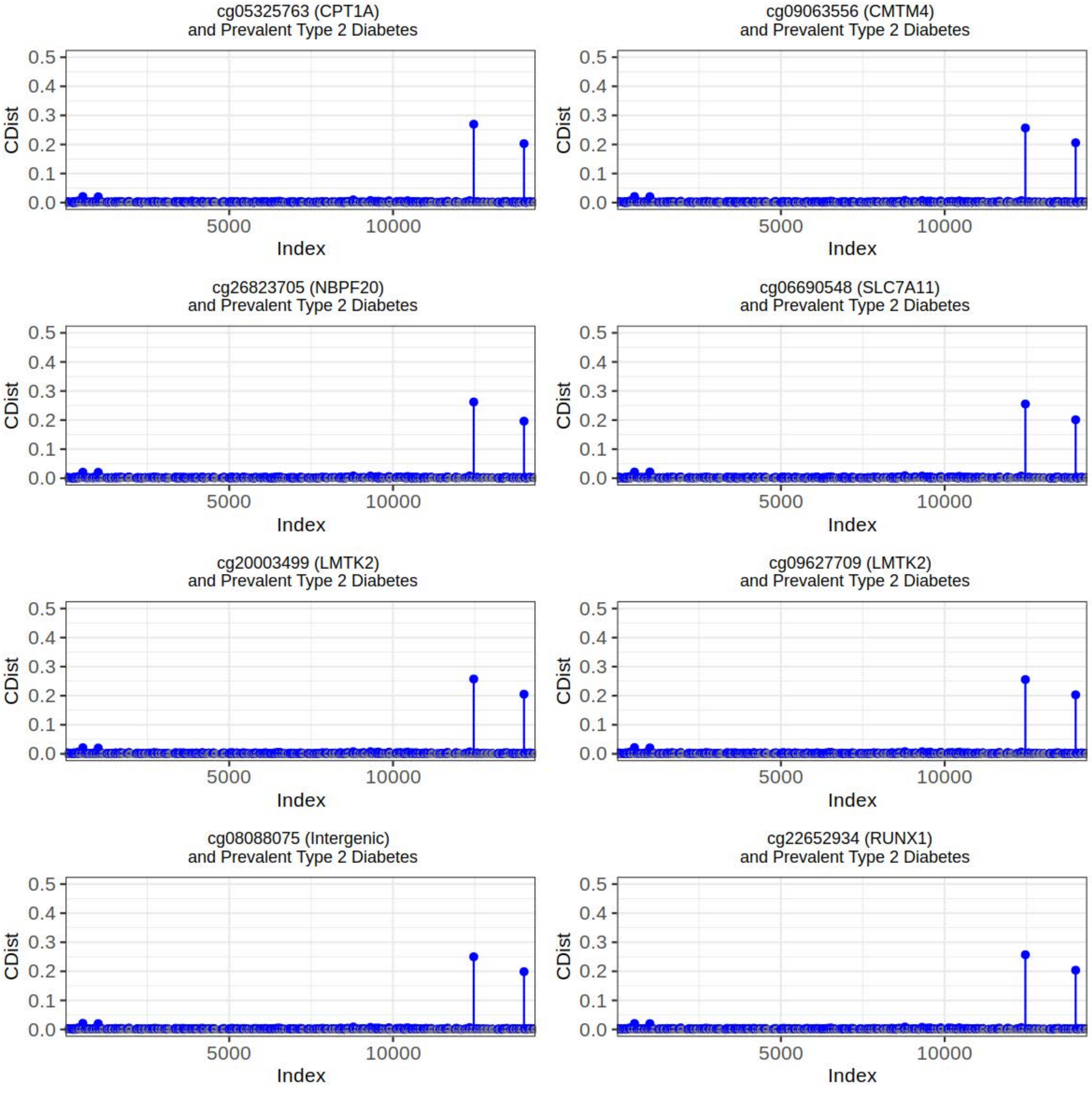
cg05325763 (CPT1A)
and Prevalent Type 2 Diabetes
cg09063556 (CMTM4)
and Prevalent Type 2 Diabetes
cg26823705 (NBPF20)
and Prevalent Type 2 Diabetes
cg06690548 (SLC7A11)
and Prevalent Type 2 Diabetes
cg20003499 (LMTK2)
and Prevalent Type 2 Diabetes
cg09627709 (LMTK2)
and Prevalent Type 2 Diabetes
cg08088075 (Intergenic)
and Prevalent Type 2 Diabetes
cg22652934 (RUNX1)
and Prevalent Type 2 Diabetes
CDist
Index
0.0
0.1
0.2
0.3
0.4
0.5
5000
10000

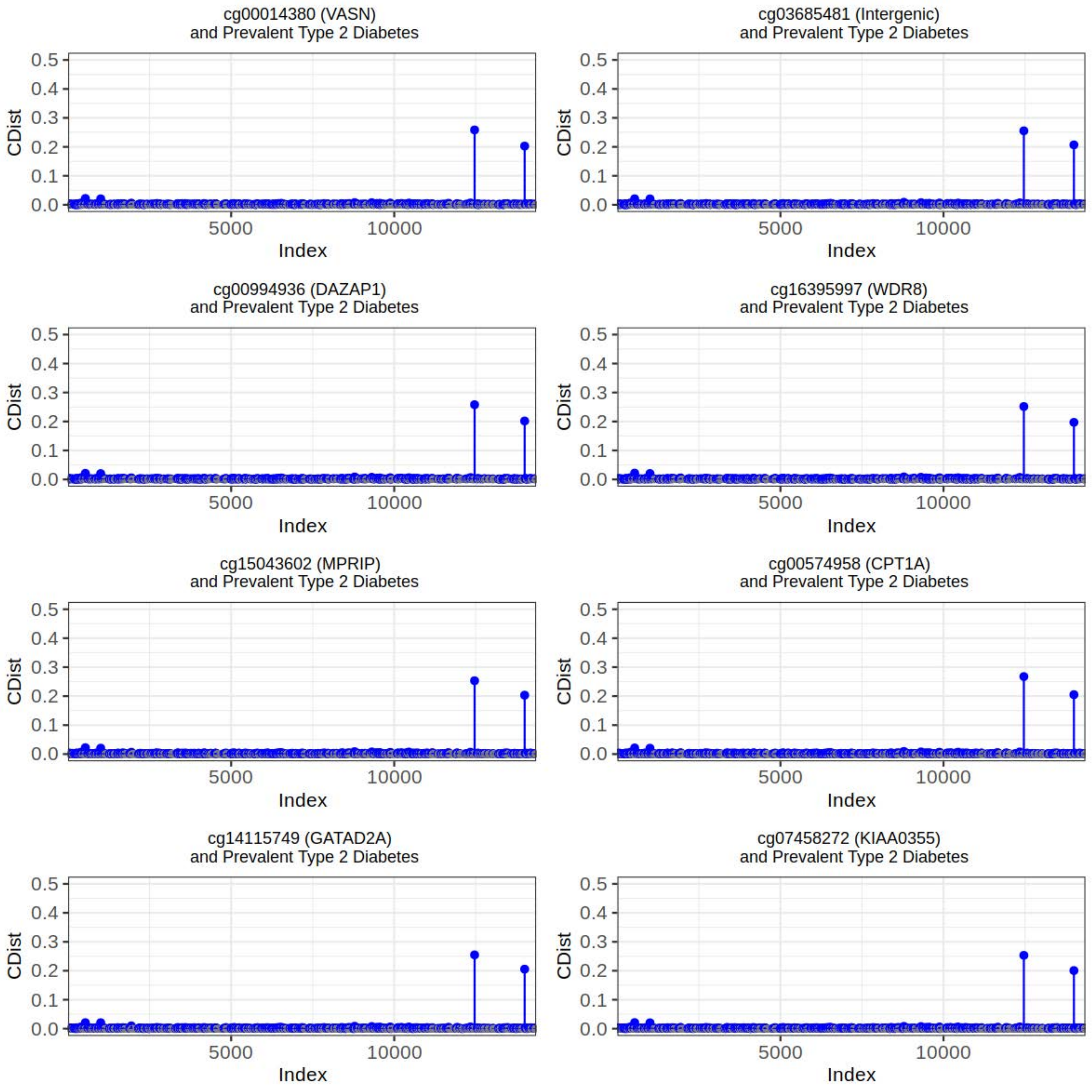
cg00014380 (VASN)
and Prevalent Type 2 Diabetes
cg03685481 (Intergenic)
and Prevalent Type 2 Diabetes
cg00994936 (DAZAP1)
and Prevalent Type 2 Diabetes
cg16395997 (WDR8)
and Prevalent Type 2 Diabetes
cg15043602 (MPRIP)
and Prevalent Type 2 Diabetes
cg00574958 (CPT1A)
and Prevalent Type 2 Diabetes
cg14115749 (GATAD2A)
and Prevalent Type 2 Diabetes
cg07458272 (KIAA0355)
and Prevalent Type 2 Diabetes
CDist
0.0
0.1
0.2
0.3
0.4
0.5
Index
5000
10000

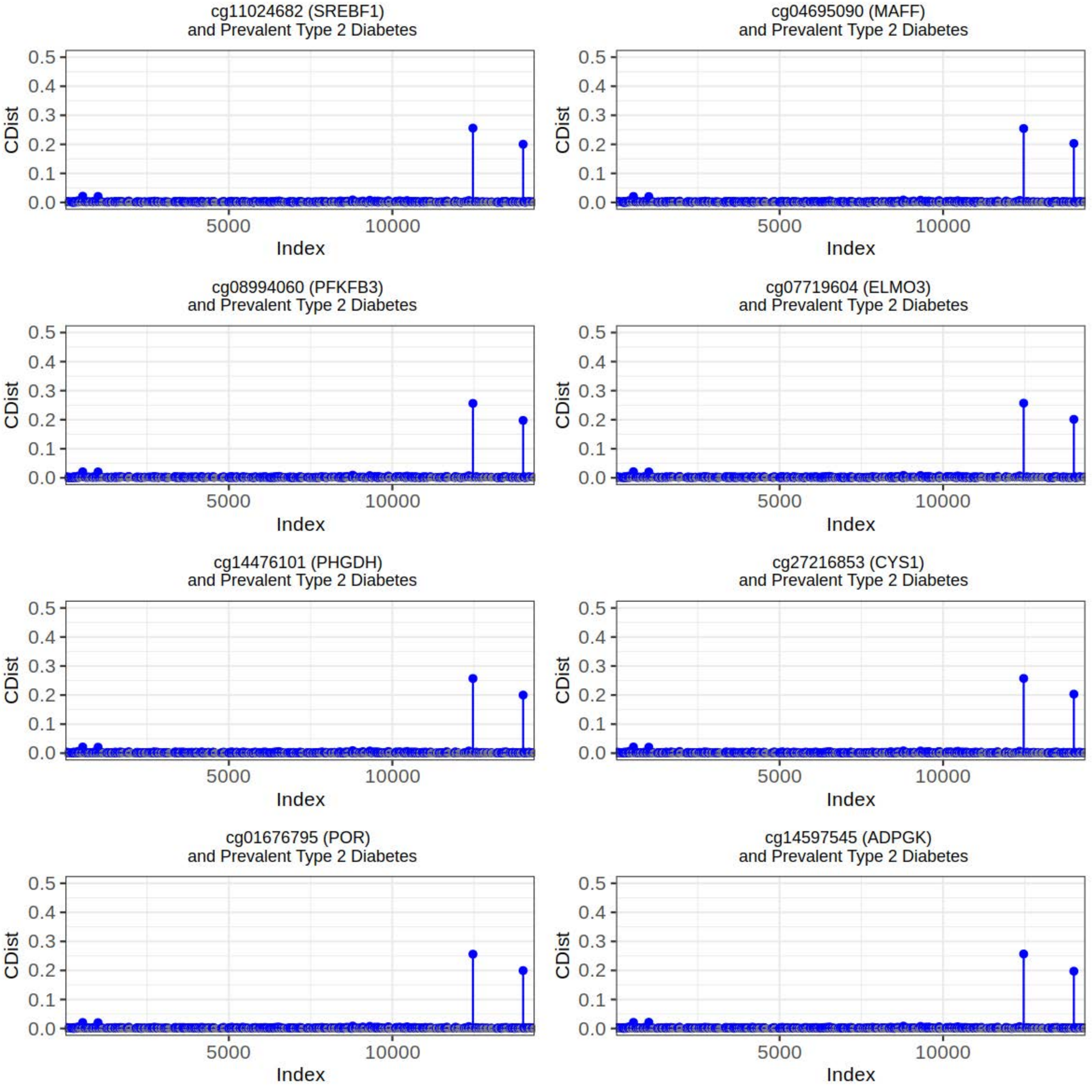
cg11024682 (SREBF1)
and Prevalent Type 2 Diabetes
cg04695090 (MAFF)
and Prevalent Type 2 Diabetes
cg08994060 (PFKFB3)
and Prevalent Type 2 Diabetes
cg07719604 (ELMO3)
and Prevalent Type 2 Diabetes
cg14476101 (PHGDH)
and Prevalent Type 2 Diabetes
cg27216853 (CYS1)
and Prevalent Type 2 Diabetes
cg01676795 (POR)
and Prevalent Type 2 Diabetes
cg14597545 (ADPGK)
and Prevalent Type 2 Diabetes
CDist
Index
0.0
0.1
0.2
0.3
0.4
0.5
5000
10000

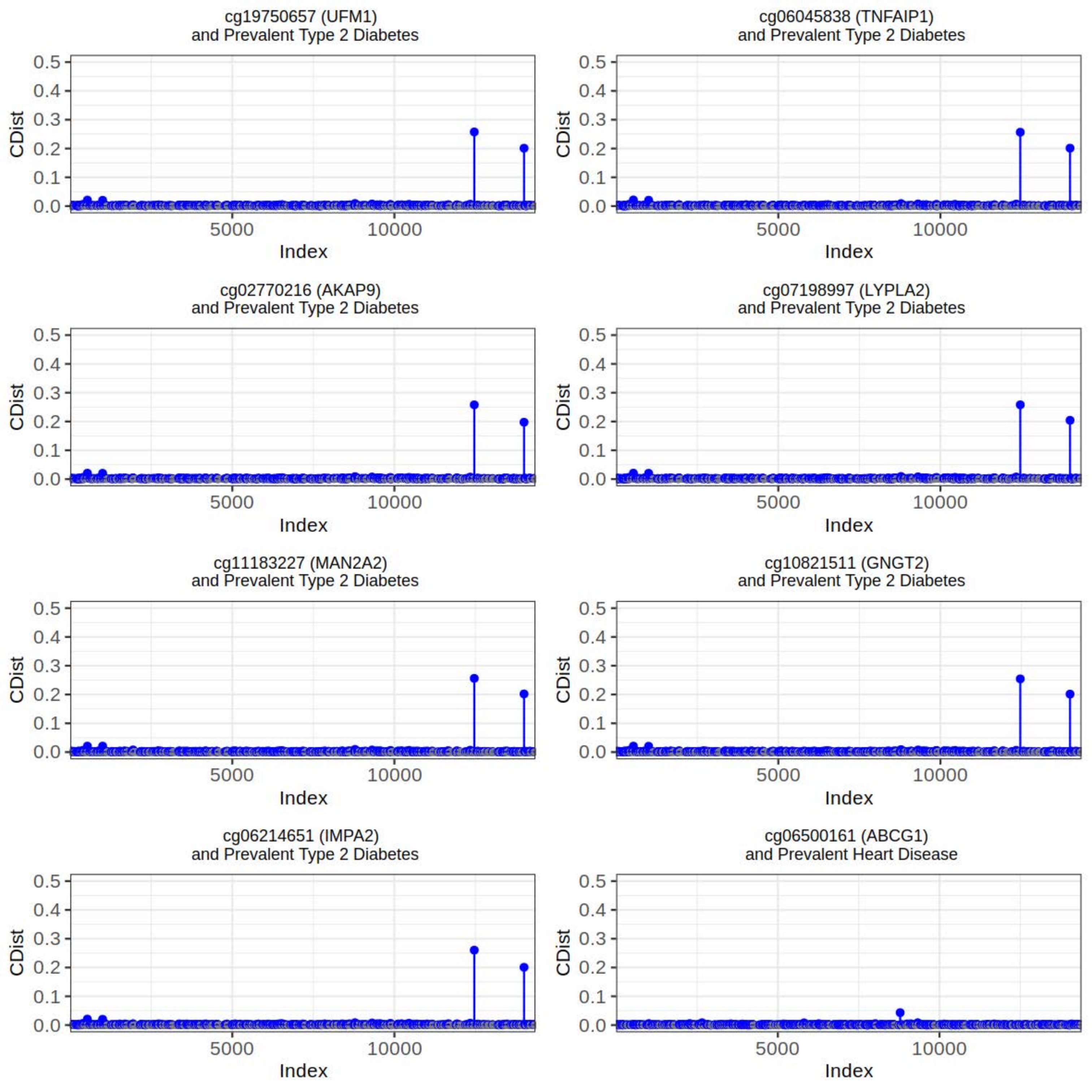
cg19750657 (UFM1)
and Prevalent Type 2 Diabetes
cg06045838 (TNFAIP1)
and Prevalent Type 2 Diabetes
cg02770216 (AKAP9)
and Prevalent Type 2 Diabetes
cg07198997 (LYPLA2)
and Prevalent Type 2 Diabetes
cg11183227 (MAN2A2)
and Prevalent Type 2 Diabetes
cg10821511 (GNGT2)
and Prevalent Type 2 Diabetes
cg06214651 (IMPA2)
and Prevalent Type 2 Diabetes
cg06500161 (ABCG1)
and Prevalent Heart Disease
CDist
Index
0.0
0.1
0.2
0.3
0.4
0.5
5000
10000

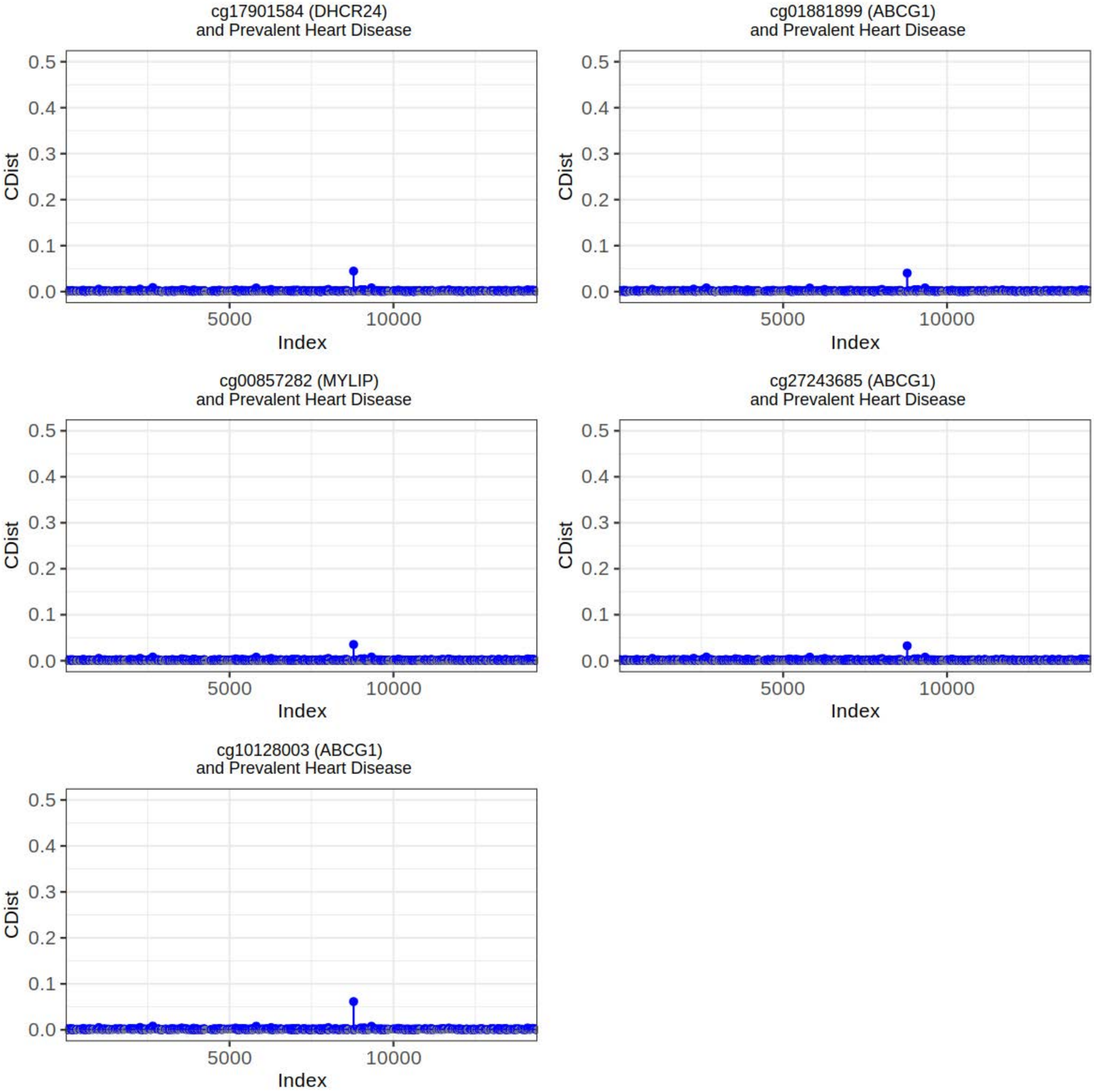
cg17901584 (DHCR24)
and Prevalent Heart Disease
CDist
0.0
0.1
0.2
0.3
0.4
0.5
5000
10000
Index
cg01881899 (ABCG1)
and Prevalent Heart Disease
CDist
Index
cg00857282 (MYLIP)
and Prevalent Heart Disease
CDist
Index
cg27243685 (ABCG1)
and Prevalent Heart Disease
CDist
Index
cg10128003 (ABCG1)
and Prevalent Heart Disease
CDist
Index
