# Supplementary material for: Blood-based epigenome-wide analyses of 19 common disease states: A longitudinal, population-based linked cohort study of 18,413 Scottish individuals
Source: PLoS Med. 2023 Jul 6;20(7):e1004247. doi: 10.1371/journal.pmed.1004247 (PMC10325072; doi:10.1371/journal.pmed.1004247)
Supplement: S1 Text — (DOCX) [file pmed.1004247.s004.docx]

**Supplementary methods for methylation quality control**

Set 1 followed a slightly different quality control strategy to Sets 2 and 3, which together followed the same strategy. In Set 1, samples were removed if: (i) ≥1% of probes had a detection *p-*value >0.05 or (ii) there was a disagreement between self-reported sex and methylation-predicted sex. Probes were removed if: (i) ≥5% of samples had a bead count <3 or a detection *p-*value >0.05, (ii) they were non-autosomal or (iii) they overlay any SNPs and/or resided in potential cross-hybridising locations [1]. In Sets 2 and 3, samples were removed if: (i) ≥0.5% of probes had a detection *p*-value >0.01 or (ii) there was a disagreement between self-reported sex and methylation-predicted sex. Probes were excluded if (i) ≥5% of samples had a bead count of 3 or less, (ii) ≥1% of samples had a detection *p*-value >0.01, (iii) they were non-autosomal or (iii) they overlay any SNPs and/or resided in potential cross-hybridising locations.

**References**

1. McCartney DL, Walker RM, Morris SW, McIntosh AM, Porteous DJ, Evans KL. Identification of polymorphic and off-target probe binding sites on the Illumina Infinium MethylationEPIC BeadChip. Genomics Data. 2016;9:22-4. doi: <https://doi.org/10.1016/j.gdata.2016.05.012>.
