# Supplementary material for: Blood-based epigenome-wide analyses of 19 common disease states: A longitudinal, population-based linked cohort study of 18,413 Scottish individuals
Source: PLoS Med. 2023 Jul 6;20(7):e1004247. doi: 10.1371/journal.pmed.1004247 (PMC10325072; doi:10.1371/journal.pmed.1004247)
Supplement: S2 Text — (DOCX) [file pmed.1004247.s005.docx]

**Supplementary methods for preparation of phenotypes**

Where possible, disease states were included in both prevalent (cross-sectional) and incident (longitudinal) analyses. However, not all of the 19 disease states were present on self-report questionnaires at Generation Scotland (GS) baseline. We used self-report data on twelve of the 19 disease states for our cross-sectional analyses. The remaining seven conditions that lacked appropriate self-report data at baseline were Alzheimer’s dementia (AD), chronic kidney disease (CKD), inflammatory bowel disease, liver cirrhosis, ovarian cancer and both COVID-19 phenotypes (i.e. severity and long COVID). We instead used self-reported parental history of AD as a proxy variable for prevalent AD owing to the age profile of our cohort (i.e. predominantly mid-life) which is justified by the near-unit genetic correlation between family history of AD and late-onset AD [1]. Furthermore, we estimated glomerular filtration rate (eGFR) from serum creatinine levels using the CKD-EPI equation and inferred CKD prevalence from the resultant data [2]. Individuals with an eGFR <60 ml/min/1.73 m^2^ were considered to have CKD.

We considered two COVID-19 outcomes: severity within those who had COVID and long COVID. COVID severity was defined as a binary outcome indicating whether the participant had been hospitalised from COVID as of October 2022. Secondary SMR01 records were used to obtain COVID-19 hospital admissions using ICD-10 codes U07.1 (lab-confirmed COVID-19 diagnosis), and U07.2 (clinically diagnosed COVID-19). Long COVID was also a binary outcome but was self-reported and obtained from a subset of GS participants who took part in the CovidLife study [3]. Long COVID was defined as self-reported symptom duration >4 weeks following first infection. Whereas COVID diagnosis was ascertained via record linkage for the severity phenotype, self-reported COVID diagnosis was considered sufficient for the long COVID phenotype to ensure that consistent data sources were used to build each phenotype.

**References**

1. Marioni RE, Harris SE, Zhang Q, McRae AF, Hagenaars SP, Hill WD, et al. GWAS on family history of Alzheimer’s disease. Translational Psychiatry. 2018;8(1):99. doi: 10.1038/s41398-018-0150-6.

2. Levey AS, Stevens LA, Schmid CH, Zhang YL, Castro AF, 3rd, Feldman HI, et al. A new equation to estimate glomerular filtration rate. Annals of internal medicine. 2009;150(9):604-12. Epub 2009/05/06. doi: 10.7326/0003-4819-150-9-200905050-00006. PubMed PMID: 19414839; PubMed Central PMCID: PMCPMC2763564.

3. Fawns-Ritchie C, Altschul DM, Campbell A, Huggins C, Nangle C, Dawson R, et al. CovidLife: a resource to understand mental health, well-being and behaviour during the COVID-19 pandemic in the UK. Wellcome Open Research. 2021;6(176):176.
