# Supplementary material for: Blood-based epigenome-wide analyses of 19 common disease states: A longitudinal, population-based linked cohort study of 18,413 Scottish individuals
Source: PLoS Med. 2023 Jul 6;20(7):e1004247. doi: 10.1371/journal.pmed.1004247 (PMC10325072; doi:10.1371/journal.pmed.1004247)
Supplement: S3 Text — (DOCX) [file pmed.1004247.s006.docx]

**Supplementary methods for sensitivity EWAS**

The proportional hazard assumption was tested using the cox.zph function in the *survival* package (global tests and local test for CpG sites) [1]. Sensitivity EWAS were performed for significant associations to further account for family structure (i.e. relatedness). This was performed for prevalent and incident disease states, considering only those associations common to basic and fully-adjusted models. These sensitivity analyses were performed using linear mixed-effects models (for prevalent disease) or mixed-effects Cox models (for incident disease) using the lmekin and coxme functions from the R *coxme* package, respectively (version 2.2-16) [2]. The same covariate strategy as the fully-adjusted stage was applied with the addition of a kinship matrix to account for relatedness.

**References**

1. Therneau TM, Lumley T. Package ‘survival’. R Top Doc. 2015;128(10):28-33.

2. Therneau T. coxme: Mixed effects Cox models. R package version 2.2–16. 2020. 2021.
