# Supplementary material for: Blood-based epigenome-wide analyses of 19 common disease states: A longitudinal, population-based linked cohort study of 18,413 Scottish individuals
Source: PLoS Med. 2023 Jul 6;20(7):e1004247. doi: 10.1371/journal.pmed.1004247 (PMC10325072; doi:10.1371/journal.pmed.1004247)
Supplement: S4 Text — (DOCX) [file pmed.1004247.s007.docx]

**Supplementary methods for methylation QTL analyses**

The GoDMC resource examined CpG sites on the Illumina 450k array and not the EPIC array, meaning some of the CpG sites in our study lack methylation or mQTL summary statistics in GoDMC. For these sites, we used mixed linear models in fastGWA to identify mQTLs in GS (n=18,413) [1]. mQTL analyses were adjusted for age, sex and batch.

**References**

1. Jiang L, Zheng Z, Qi T, Kemper KE, Wray NR, Visscher PM, et al. A resource-efficient tool for mixed model association analysis of large-scale data. Nature Genetics. 2019;51(12):1749-55. doi: 10.1038/s41588-019-0530-8.
