# Supplementary material for: Blood-based epigenome-wide analyses of 19 common disease states: A longitudinal, population-based linked cohort study of 18,413 Scottish individuals
Source: PLoS Med. 2023 Jul 6;20(7):e1004247. doi: 10.1371/journal.pmed.1004247 (PMC10325072; doi:10.1371/journal.pmed.1004247)
Supplement: S5 Text — (DOCX) [file pmed.1004247.s008.docx]

**Supplementary note on covariate-specific attenuation of effect sizes in basic model**

We examined the contribution of each of the five common lifestyle risk factors in attenuating the 1,340 prevalent associations and 14,237 incident associations that were brought forward to the fully adjusted model. We repeated each model including only one of these five covariates at a time and calculated the percentage by which the effect size changed from that of the basic model. As outlined in the main text, the risk factors were alcohol consumption, body mass index, deprivation, education and a methylation-based surrogate for smoking behaviour. The mean attenuation in effect sizes by each of the covariates ranged from 5.5% (for body mass index) to 63.1% (for smoking). However, there was heterogeneity across disease states given their distinct risk profiles. For instance, body mass index induced the strongest attenuation for associations involving type 2 diabetes (mean reduction of 31.7%) whereas smoking showed a mean attenuation of 11.2% for this trait. By contrast, smoking attenuated effect sizes for COPD associations, on average, by 78.6% (**S14 Table**). The high degree of attenuation by smoking across all associations is likely attributed to COPD possessing the majority of associations in the basic model (>75%).
