# Supplementary material for: Blood-based epigenome-wide analyses of 19 common disease states: A longitudinal, population-based linked cohort study of 18,413 Scottish individuals
Source: PLoS Med. 2023 Jul 6;20(7):e1004247. doi: 10.1371/journal.pmed.1004247 (PMC10325072; doi:10.1371/journal.pmed.1004247)
Supplement: S1 Fig — (DOCX) [file pmed.1004247.s009.docx]

**S1 Fig. Associations between covariates and prevalent disease states in univariable and multivariable logistic regression models.**


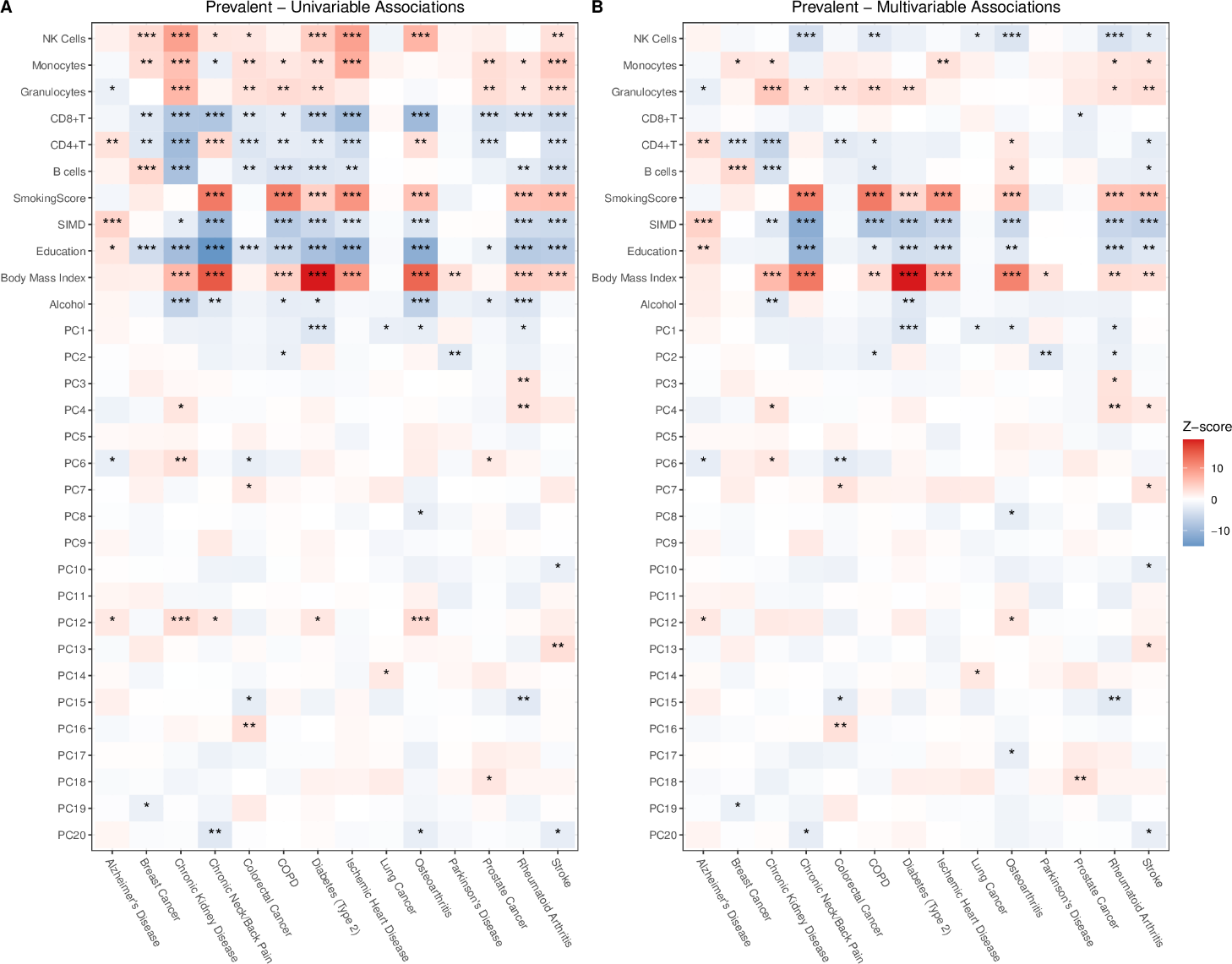


Univariable models considered only the covariate and disease state as a binary outcome. Multivariable models were additionally adjusted for age and sex to obtain regression coefficients. These data are graphical representations of the data shown in **S4 Text**. Granulocytes are shown for completeness despite being excluded from models on the basis of collinearity. Stars denote the following levels of significance: *, *p<*0.05; **, *p<*0.01; ***, *p<*0.001. SIMD, Scottish Index of Multiple Deprivation.
