# Supplementary material for: Blood-based epigenome-wide analyses of 19 common disease states: A longitudinal, population-based linked cohort study of 18,413 Scottish individuals
Source: PLoS Med. 2023 Jul 6;20(7):e1004247. doi: 10.1371/journal.pmed.1004247 (PMC10325072; doi:10.1371/journal.pmed.1004247)
Supplement: S2 Fig — (DOCX) [file pmed.1004247.s010.docx]

**Fig S2. Associations between covariates and incident disease states in univariable and multivariable Cox proportional hazards models.**


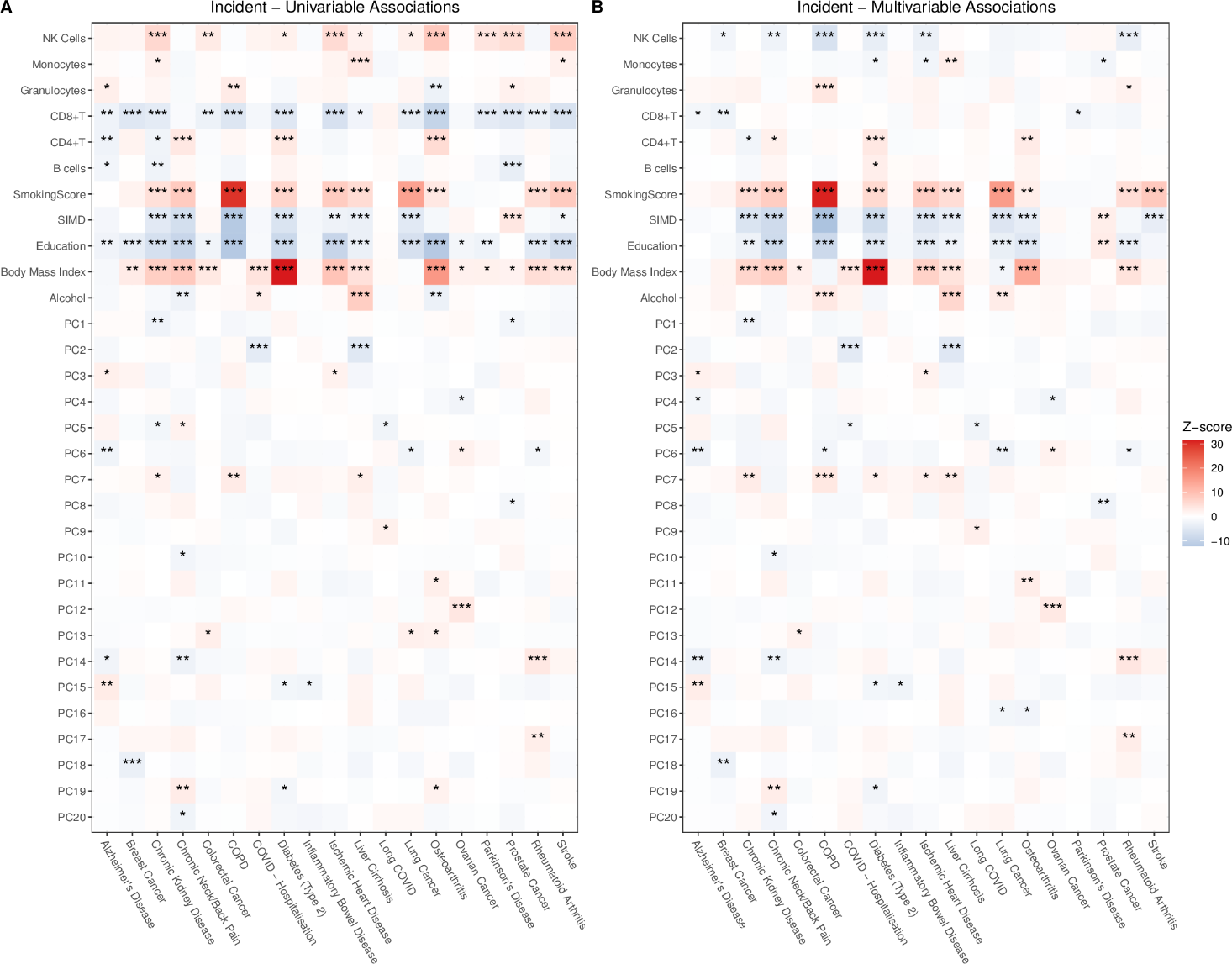
 Univariable models considered only the covariate and disease state. Multivariable models were additionally adjusted for age and sex to obtain regression coefficients. These data are graphical representations of the data shown in **S5 Table**. Granulocytes are shown for completeness despite being excluded from models on the basis of collinearity. Stars denote the following levels of significance: *, *p<*0.05; **, *p<*0.01; ***, *p<*0.001. SIMD, Scottish Index of Multiple Deprivation.
