# Supplementary material for: Blood-based epigenome-wide analyses of 19 common disease states: A longitudinal, population-based linked cohort study of 18,413 Scottish individuals
Source: PLoS Med. 2023 Jul 6;20(7):e1004247. doi: 10.1371/journal.pmed.1004247 (PMC10325072; doi:10.1371/journal.pmed.1004247)
Supplement: S3 Fig — (DOCX) [file pmed.1004247.s011.docx]

**S3 Fig. Correlation between effect sizes from linear regression EWAS and sensitivity linear mixed effects analyses that further accounted for relatedness.**


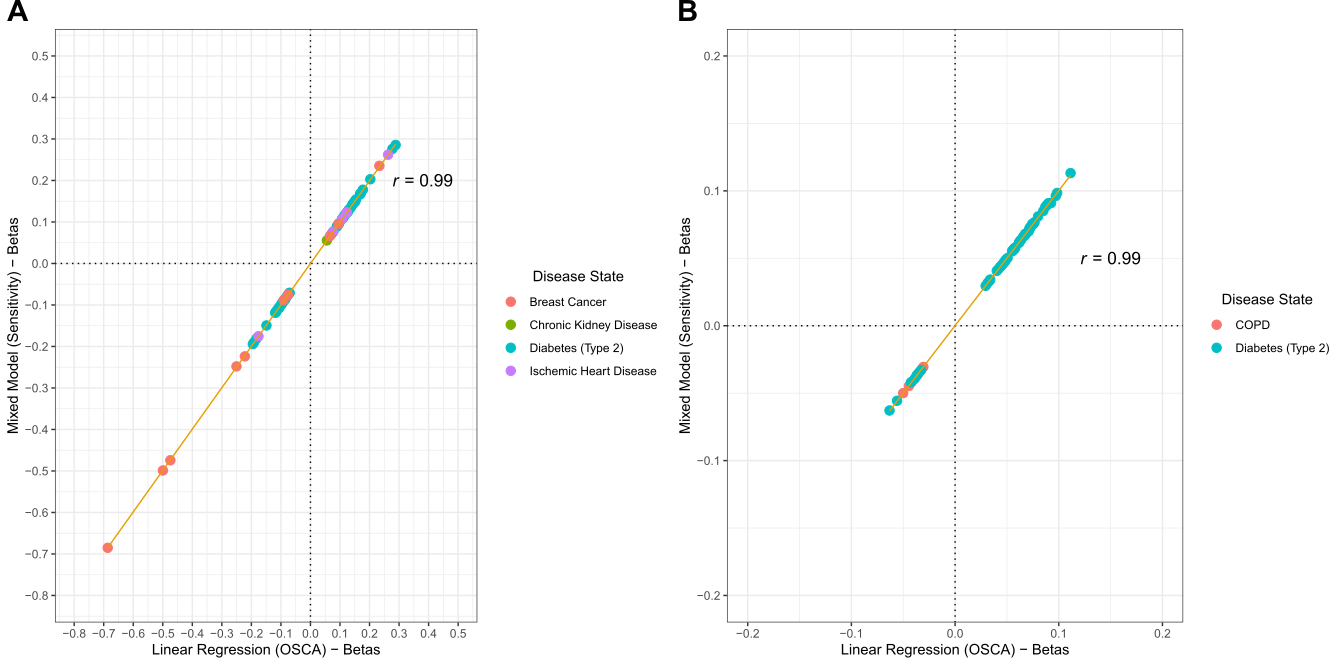


There was an almost unit correlation between effect sizes from the standard EWAS approach that included related individuals in Generation Scotland and effect sizes from mixed effects models that accounted for relatedness. Associations represent those that were common to both basic and fully-adjusted models. Disease states are annotated for prevalent (A) and incident disease (B) separately. COPD, chronic obstructive pulmonary disease; EWAS, epigenome-wide association study.
