# Supplementary material for: Blood-based epigenome-wide analyses of 19 common disease states: A longitudinal, population-based linked cohort study of 18,413 Scottish individuals
Source: PLoS Med. 2023 Jul 6;20(7):e1004247. doi: 10.1371/journal.pmed.1004247 (PMC10325072; doi:10.1371/journal.pmed.1004247)
Supplement: Attachment — Submitted filename: hillary Plos medicine 23.docx [file pmed.1004247.s037.docx]

This study seeks to identify epigenetic signals of disease prevalence or incidence in the Generation Scotland study. The paper is concisely written and reports some novel EWAS which is a significant contribution to the existing literature. A particularly interesting feature of the paper is the effort to examine concordance of previous EWAS results but this needs to be strengthened to help the reader gain an informed insight into what these results show.

The concordance between the EWAS in GS and previous literature is not currently very detailed. For example, it is not clear what the authors accept as replication between studies – for disease states where there is a number of EWAS (eg T2D) are they accepting CpG sites that are replicated across all studies or do they accept any CpG that is reported in 2 or more studies? Do the authors consider direction of effect or potential heterogeneity across genetic ancestries (although most studies are predominantly European ancestry)? Do they consider the statistical models used in different EWAS? What is the replication in studies using GS data eg Bermingham et al PMID 30935889?

The information being reported here is of consequence as it tells us if EWAS is an avenue of research worth following at all – if concordance between studies is very low and it is not explainable it suggests there is a problem in running these kinds of studies. It would also help if this was put into context – how much replication do we see between well powered GWAS studies? The other comment here is that the authors do not seem to present an appraisal of overlap amongst previous studies on the same disease phenotypes even though they say this will be presented in the introduction.

I have some minor areas needing clarification or improvement:

In 2.4 the adjustment for age, sex and batch could be more clearly described. How are age/sex/batch adjusted M values generated? What are the batches? The batch correction is likely to be incomplete if only one batch factor was included and this needs to be acknowledged. Other commonly used approaches such as SVA may not be feasible in this analysis model but the authors need to justify their approach as it may impact how they interpret replication with other studies.

In the fully adjusted model, can the authors justify their model choice? For example, is adjustment for BMI appropriate in T2D model?

For the mQTL analyses, did the authors check if the mQTL associations were similar between GS and GoDMC for the CpGs that were present on the 450k array? This would give an estimate of how well powered GS is to detect mQTLs within GS and whether there is substantial heterogeneity between GS and GoDMC mQTLs.

Figure 5 is very difficult to interpret – For example in group 4 (EPIC array, n=2) is this prevalent and/or incident disease? For replication with existing studies is “genes replicate” any CpGs at an annotated locus or something else? For “Replication of our study with existing studies” which of the numerator /denominator are from the current study? Why is only CKD and T2D in this box?

The study lists a number of limitations but these could be discussed in more detail if word count constraints allowed. For example, discuss case ascertainment issues of using parental history of AD as a proxy for variable for AD. The discussion of replication between studies could also be discussed in more detail if space allowed.
